# Physiological Responses of Young Pea and Barley Seedlings to Plasma-Activated Water

**DOI:** 10.3390/plants10081750

**Published:** 2021-08-23

**Authors:** Dominik Kostoláni, Gervais B. Ndiffo Yemeli, Renáta Švubová, Stanislav Kyzek, Zdenko Machala

**Affiliations:** 1 Department of Plant Physiology, Faculty of Natural Sciences, Comenius University in Bratislava, Mlynská Dolina, Ilkovičova 6, 842 15 Bratislava, Slovakia; kostolani6@uniba.sk (D.K.); renata.svubova@uniba.sk (R.Š.); 2 Division of Environmental Physics, Faculty of Mathematics, Physics and Informatics, Comenius University in Bratislava, Mlynská Dolina, 842 48 Bratislava, Slovakia; gbn.yemeli@fmph.uniba.sk; 3 Department of Genetics, Faculty of Natural Sciences, Comenius University in Bratislava, Mlynská Dolina, Ilkovičova 6, 842 15 Bratislava, Slovakia; kyzek2@uniba.sk

**Keywords:** antioxidant enzymes, barley, non-thermal plasmas, dehydrogenases, DNA damage, lytic enzymes, pea, plasma-activated water, RONS

## Abstract

This study demonstrates the indirect effects of non-thermal ambient air plasmas (NTP) on seed germination and plant growth. It investigates the effect of plasma-activated water (PAW) on 3-day-old seedlings of two important farm plants—barley and pea. Applying different types of PAW on pea seedlings exhibited stimulation of amylase activity and had no inhibition of seed germination, total protein concentration or protease activity. Moreover, PAW caused no or only moderate oxidative stress that was in most cases effectively alleviated by antioxidant enzymes and proved by in situ visualization of H_2_O_2_ and ˙O_2_^−^. In pea seedlings, we observed a faster turn-over from anaerobic to aerobic metabolism proved by inhibition of alcohol dehydrogenase (ADH) activity. Additionally, reactive oxygen/nitrogen species contained in PAW did not affect the DNA integrity. On the other hand, the high level of DNA damage in barley together with the reduced root and shoot length and amylase activity was attributed to the oxidative stress caused by PAW, which was exhibited by the enhanced activity of guaiacol peroxidase or ADH. Our results show the glow discharge PAW at 1 min activation time as the most promising for pea. However, determining the beneficial type of PAW for barley requires further investigation.

## 1. Introduction

In light of the rapidly increasing world population, a demand for sustainable food production becomes more and more critical [1]. Despite many efforts, the sufficient agricultural production generating high-quality crops depends on the use of commercial fertilizers. From all micro- and macronutrients contained in fertilizers, nitrogen represents the main compound promoting plant growth and production [2,3]. Considering a negative impact of chemicals on human and animal health and the environment, the recent research focuses on alternative ways to promote plant growth which are less harmful, more sustainable and efficient [3,4]. Besides organic fertilizers and biofertilizers, which also pose some risks and/or have limitations [5,6], physical methods such as static magnetic field or pulsed electric field indicate promising results [7,8]. The recent use of cold atmospheric gas plasmas is also considered promising but demands further investigations to understand the mechanisms of interactions with plants.

Plasma represents the fourth state of matter and occurs naturally in space or Earth’s atmosphere or can be produced artificially under laboratory conditions [9,10]. According to its temperature and thermal equilibrium of charged and neutral particles, plasma can be categorised as thermal or non-thermal (cold, nonequilibrium) plasma [11]. Unlike in thermal plasma, the excitation, dissociation or ionization of molecules in non-thermal plasma (NTP) occurs with higher effectiveness and causes significantly lower temperature of heavy particles when compared to the electron temperature. Relatively low temperature of NTP allows its subsequent application for the treatment of thermo-sensitive and biological materials [11,12,13,14].

The application of NTP in agriculture includes both a direct and indirect way of plasma treatment. A major part of published data showed the effects of the direct way that represents an explicit interaction between the plasma and the plant material. This method has been approved in seed sterilization, germination enhancement and plant growth promotion [15,16,17,18,19,20,21,22,23]. Far less studies including this article have been focused on the indirect plasma treatment, where the interaction between plasma and plant material is mediated by plasma-activated water (PAW) [24,25,26,27,28]. The PAW is usually generated by performing the electrical discharges directly in water or on its surface [29,30,31]. Regarding the type of plasma discharge, technique of interaction with water and water solution buffering capacity, various types of PAW may strongly differentiate in chemical composition, especially in concentration of long-lifetime reactive oxygen and nitrogen species (RONS) delivered by the interaction with the plasma, such as hydrogen peroxide (H_2_O_2_), nitrites (NO_2_^−^) and nitrates (NO_3_^−^) [32].

Hydrogen peroxide can easily enter the plant cells via free diffusion or via water channels aquaporins. As the most stable reactive oxygen species, it plays a crucial role in the cascades of intracellular signalling. However, if abundant, it can be metabolised to water and oxygen by the catalase and peroxidase activities [21,33]. The NO_2_^−^ and particularly NO_3_^−^ contained in PAW represent an important source of nitrogen for plants, and their transport across the plasma membrane is provided via diffusion or via specific transporters [34,35]. Thanks to its composition, PAW has the potential to become an environment-friendly and sustainable alternative to classical fertilizers used in agriculture.

Maniruzzaman [36] documented the enhanced effect of PAW RONS on plant growth in comparison with chemical fertilizers. The study referred better results to long-life RONS in PAW, which can be in the solution with chemical fertilizers partially replaced by unstable intermediate species. Gierczik et al. [37] showed that pre-treatment of barley grains increased germination under abiotic stress through the improved signalling and activation of different defence mechanisms by H_2_O_2_ and nitric oxide (NO; formed from NO_2_^−^ and NO_3_^−^). Nevertheless, a wide range of plasma sources and plant samples tested in scattered studies and resulting in different efficiencies of treatment requires further investigations.

The objective of this article is to study the influence of PAW generated by two sources of cold atmospheric air plasma, namely transient spark (TS) with water electrospray and glow discharge (GD) with water cathode on pea (*Pisum sativum* L. cv. Eso) seeds and barley (*Hordeum vulgare* L. cv. Kangoo) grains. Before applying PAW to the plants, we measured the concentration of long-life species (H_2_O_2_, NO_2_^−^, NO_3_^−^) in various types of PAW by UV–Vis absorption spectroscopy. Then, we analysed specific plant growth parameters (germination, root, shoot and seedling length) and physiological parameters, such as total soluble proteins concentration (TSP) and DNA damage. Since the RONS are the main monitored components in PAW, we also focused on antioxidant enzyme activities (superoxide dismutase [SOD], guaiacol peroxidase [G-POX] and catalase [CAT]) and in situ visualization of H_2_O_2_ and ˙O_2_^−^. Finally, the activity of lytic enzymes (proteases, amylases) and dehydrogenases (alcohol and succinate dehydrogenases) were determined. A lack of studies investigating the activity of these enzymes after PAW treatment in relation to other growth and physiological parameters in early stages of plant development represents the novelty of this part of the study. Hopefully, our results will contribute to the better understanding of relations between certain plasma treatments and plant species, resulting in changes in the plant metabolism and plant growth.

## 2. Results and Discussion

### 2.1. Physiochemical Properties of PAW

**Figure 1:**
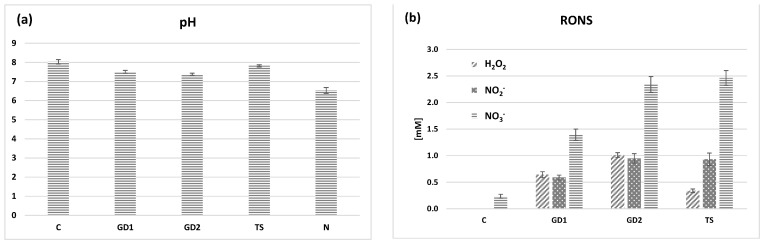
pH (**a**) and concentrations of hydrogen peroxide (H_2_O_2_), nitrite (NO_2_^−^) and nitrate (NO_3_^−^) (**b**) in tap water control (C), PAW generated by glow discharges for 1 and 2 min (GD1 and GD2, respectively), PAW generated by transient spark (TS) and 2 mM of nitric acid (N). Values are expressed as a mean ± SD, minimum ten repetitions.

Figure 1a shows the pH of the tap water control and PAW and 2 mM HNO_3_. A slight decrease of pH from 8 to 7.8 was found between the control and TS PAW as well as from 7.5 and 7.4 for GD1 (PAW of glow discharge at activation time 1 min) and GD2 (PAW of glow discharge at activation time 2 min), respectively. The small difference of pH before and after the plasma treatment can be explained by the natural hydrocarbon buffer system, unlike when plasma treating deionized or distilled water where we observed a strong acidification [38,39,40]. Our previous articles also reported these slight pH changes using tap water [21,26,28]. It turns out that overall pH variations are negligible, making this parameter a non-disruptive factor of the germination process.

Figure 1b shows the concentrations of H_2_O_2_, NO_2_^−^ and NO_3_^−^ generated in the three types of PAW generated by both plasma sources. The following concentrations were obtained for H_2_O_2_ ~0.33 mM, 0.64 mM and 1.00 mM for TS, GD1 and GD2, respectively. The concentrations of NO_2_^−^ for TS, GD1 and GD2 were approximately 0.93 mM, 0.59 mM and 0.95 mM, respectively. The concentrations of NO_3_^−^ for TS, GD1 and GD2 were approximately 2.46 mM, 1.40 mM and 2.34 mM, respectively. Both plasma sources are rich providers of RONS in water, as previously shown [28,38,39,41]. Thanks to the almost constant pH, these RONS do not decay after plasma treatment over a period of several hours, which facilitates their application on plants.

### 2.2. Germination Dynamics

**Figure 2:**
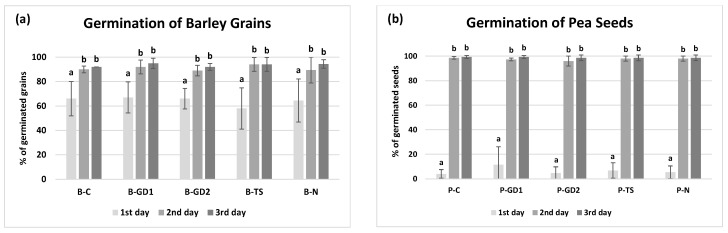
The percentage of barley grain (**a**) and pea seed (**b**) germination in 3-day horizon, watered with tap water (control: B/P-C), plasma-activated water (PAW) of glow discharge with water cathode and treatment time 1 and 2 min (B/P-GD1 and B/P-GD2), PAW of transient spark discharge with electrospray (B/P-TS) and 2-mM nitric acid (B/P-N). Values are expressed as a mean ± SD from three repeated experimental rounds (one run represents 50 grains/seeds per variant, n = 150). Different letters represent statistically significant difference at *p* < 0.05 according to LSD test.

The three types of plasma-activated water (PAW) have a slight impact on the percentage of germination (%) or germination dynamics (%) of barley grains and pea seeds (Figure 2). This is probably because the seeds/grains used in our study retained almost 100% germination rate even under control conditions. In the case of barley grains, we observed a slight decrease in the percentage of germination (%) in the variant B-TS on the first day of cultivation, but on the third day, the number of germinated grains was comparable to other variants (Figure 2a). The highest germination, compared to the control (89%), was recorded in the case of the B-GD1 variant (96%). Pea seeds started the germination processes most effectively in the case of the P-GD1 variant (first day of cultivation). Already on the second day of cultivation, germination levelled for all variants and reached approximately 98% (Figure 2b). In published studies, fairly contrast results considering PAW’s effect on the germination can be found. These results suggest, on one hand, a stimulative effect, as shown for example by Kučerová et al. [26] on wheat plants, Zhang et al. [42] on lentils with an increased germination rate of about 50%, or Zhou et al. [25] on mung beans with the observed shortening of the germination time up to 36 h when compared to untreated plants. In these studies, authors referred the positive effect of PAW to the H_2_O_2_ ability to stimulate the respiration, react with germination inhibitors and cause erosion of seed coat, facilitating an improved imbibition, as confirmed also by other studies [43,44]. Moreover, reactive nitrogen species in PAW may be involved in intracellular signalling and interactions with endogenous phytohormones either by regulating abscisic acid concentration or by stimulating of gibberellic acid synthesis [45]. On the other hand, Lindsay et al. [46] found no significant changes in the germination rate of radishes, tomatoes and marigolds irrigated with PAW. However, during the growth phase, there were significant differences between treated and untreated plants. These results compared to ours indicate that PAW application could significantly affect germination rate in seeds with naturally low germination rate or accelerate germination, but it also strongly depends on the plant species, which makes the final effect difficult to predict without an experimental backup.

### 2.3. Growth Parameters

**Figure 3:**
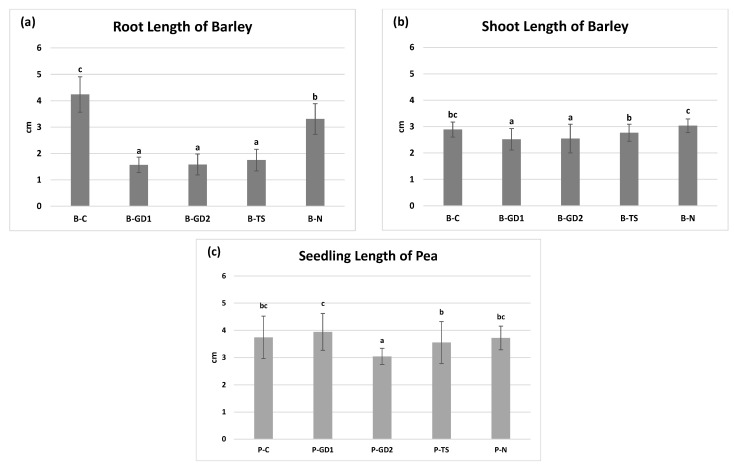
The growth parameters of 3-day-old seedlings of barley (**a**,**b**) and pea (**c**), watered with tap water (control: B/P-C), plasma-activated water (PAW) of glow discharge with water cathode and treatment time 1 and 2 min (B/P-GD1 and B/P-GD2), PAW of transient spark discharge with electrospray (B/P-TS) and 2-mM nitric acid (B/P-N). Values are expressed as a mean ± SD from five repeated experimental rounds (one run represents 10 seedlings per variant, n = 50). Different letters represent statistically significant difference at *p* < 0.05 according to LSD test.

When measuring production parameters (length of barley roots and shoots, length of pea seedlings) of 3-day-old seedlings, we found that the application of PAW and 2-mM nitric acid solution negatively affected the length of the roots of barley seedlings (Figure 3a): more than 3-fold reduction occurred for B-GD1, B-GD2 and B-TS variants and approximately one third reduction for B-N variant). It is important to mention that the number of adventitious roots was not negatively affected and shoot lengths (Figure 3b) in all treated variants were comparable to the untreated control. Shashikanthalu et al. [47] also reported changes in root length in 14-day-old *Cuminum cyminum* roots where the seeds were subjected to direct NTP treatment. In this study, seeds exposed to non-thermal plasma for 2 min and 4 min exhibited the root growth reduction of 12.6% and 8.78%, respectively. Otherwise, the seeds exposed to NTP for 3 min exhibited an increase in root length of about 41.79%. By the shoot length, they documented a positive effect under exposure times of 2 min and 3 min (20.82% and 34.5%, respectively). Longer exposure time (4 min) induced a decrease in the shoot length. On the other hand, Feizollahi et al. [48] using direct plasma treatment on barley grains documented root growth reduction in 7-day-old barley seedlings for an exposure time of 10 min, while exposure times of 1 min and 6 min caused no changes compared to control. Additionally, none of the mentioned exposure times caused a decrease in the shoot length. These findings indicate that certain exposure times (in direct plasma treatment) and different RONS contents in PAW, specific to different plant species, may be responsible for dramatic changes in growth parameters varying between roots and shoots.

In our experiments, the total length of pea seedlings in variants P-GD1 and P-TS did not change when compared to the negative and positive control (Figure 3c). On the other hand, the application of GD of 2 min caused a significant reduction in the length of pea seedlings (Figure 3c, P-GD2 variant). With respect to other results, we can conclude that pea is able to resist to the external supplementation of reactive species, without critical changes in growth parameters, more effectively than barley. Our results correspond with a study by Švubová et al. [49], who documented no or negative impact of direct NTP treatment (applicated on seeds) on 3-day-old pea seedling length under different exposure times (10–40 s) and gas atmospheres (ambient air, nitrogen and oxygen). On the contrary, a stimulative effect on the seedling length was shown by Stolárik et al. [18] when seeds were exposed to the direct plasma treatment for longer times (60 s and 180 s), possibly even regarding the effect of components other than RONS in the direct plasma treatment.

### 2.4. Total Soluble Proteins Concentration

**Figure 4:**
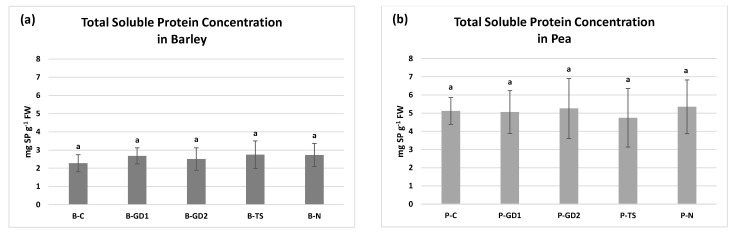
The total soluble protein concentration in the seedlings of barley (**a**) and pea (**b**) after 3 days of growth, watered with tap water (control: B/P-C), plasma-activated water (PAW) of glow discharge with water cathode and treatment time 1 and 2 min (B/P-GD1 and B/P-GD2), PAW of transient spark discharge with electrospray (B/P-TS) and 2-mM nitric acid (B/P-N). Values are shown as mean ± SD from three experimental rounds (one run represents five seedlings per variant, 1.5 g mixed samples were analysed per one experimental run and each variant for total soluble protein concentration). Different letters represent statistically significant difference at *p* < 0.05 according to LSD test.

Despite the moderate effect on growth parameters, the application of PAW and 2 mM nitric acid had no statistically significant impact on the total soluble protein (TSP) concentrations neither in barley (Figure 4a) nor in pea (Figure 4b). Almost two-fold higher values for TSP in pea (compared to barley) can be linked to different storage substances in grains and seeds of barley and pea, respectively. An increase in TSP in 4-week-old plants irrigated with PAW was documented by our preceding study by Yemeli et al. [28] for the same types of PAW in variants GD2 and N for barley and variants GD1 and GD2 for maize. Kučerová et al. [26] showed no changes in TSP in above-ground parts of wheat plants and only slightly increased concentration in roots after application of PAW generated by transient spark discharge from tap and deionized water. The effect of direct plasma exposure was also documented. Švubová et al. [49] documented an amelioration of TSP concentration under NTP treatment of pea in ambient air and oxygen with exposure times of 10 s and 40 s, respectively. On the contrary, plasmas generated in the same gases for 20 s caused a significant decrease of TSP. However, almost all combinations of gases and exposure times showed no changes in TSP concentration, as shown in the study by Švubová et al. [20]. With respect to these findings, we can state that even the same type of PAW can result in different metabolic responses when considering different plant age and application method. Additionally, it becomes even more complicated with various types of direct NTP applications, which brings additional physical effects into play (e.g., electric field, UV radiation, short-lived reactive species).

### 2.5. Activity of Lytic Enzymes

**Figure 5:**
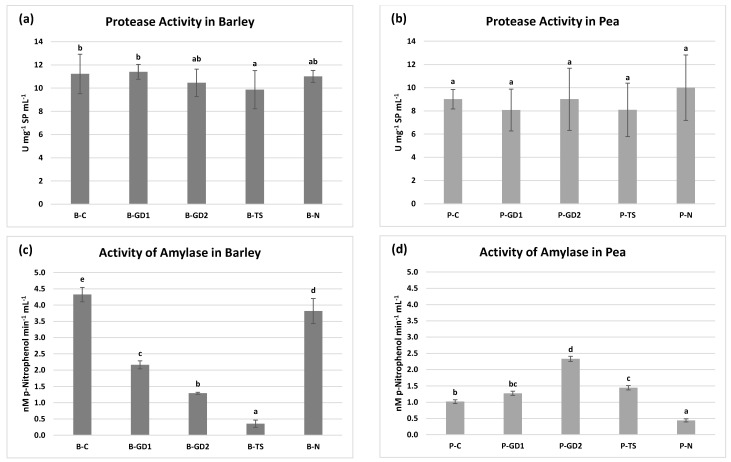
Protease and amylase activities in the barley (**a**,**c**) and pea (**b**,**d**) seedlings after 3 days of growth, watered with tap water (control: B/P-C), plasma-activated water (PAW) of glow discharge with water cathode and treatment time 1 and 2 min (B/P-GD1 and B/P-GD2), PAW of transient spark discharge with electrospray (B/P-TS) and 2-mM nitric acid (B/P-N). Values are shown as mean ± SD from three experimental rounds (one run represents five seedlings per variant, 1.5 g mixed samples were analysed per one experimental run, and each variant for protease activity and five 0.1 g mixed samples were analysed per one experimental run and each variant for amylase activity). Different letters represent statistically significant difference at *p* < 0.05 according to LSD test.

The activity of protease, the enzyme that cleaves storage proteins, was slightly affected (Figure 5a,b). In the barley B-TS variant, its activity decreased significantly. In the B-GD1 variant, we observed a slight increase in protease activity, which, however, was not statistically significant compared to the untreated control (Figure 5a). In pea, we recorded no statistically significant differences when compared to the control (Figure 5b). Unlike protease, the activity of amylase, a starch-cleaving enzyme, was strongly and negatively affected in case of barley (Figure 5c). In the B-TS variant, the amylase activity decreased by about 90% compared to the untreated seedlings. Significant reduction was also recorded in the other three variants. In pea, P-N was the only negatively affected variant (Figure 5d). On the other hand, when compared to the control, the variant P-GD2 reached more than twofold increase. Several studies linking amylase activity to proper seed development directly affecting plant growth and yield, as summarised, i.e., in the review from Damaris et al. [50].

Despite the fact that many authors attribute the increased germination to the higher activity of lytic enzymes, the pool of published data examining the effect of NTP on these enzymes is considerably restricted. Moreover, the available data are focused mainly on the direct plasma treatment. For example, Peťková et al. [51] showed a positive effect of NTP generated in three different atmospheres with short exposure time (10–30 s) on barley grains, while longer exposure time (60 s and 180 s) caused an opposite effect on protease activity. These results were also confirmed by Švubová et al. [20,49] on pea seeds. They documented a positive effect under exposure times varying from 10 s to 40 s and no or negative effect under exposure times varying from 60 s to 300 s for all tested working gases. Furthermore, higher exposure times were responsible for a significant decrease in amylase activity, with the lowest activity in the plasma-treated samples at 300 s. Sadhu et al. [52] obtained a stimulative effect of direct plasma treatment on the amylase activity of mung bean under varying exposure times and power levels. Enhanced activity of amylase in brown rice grains was also documented by Chen et al. [53] after using direct plasma treatment. Regarding this knowledge based on direct NTP effects, we can assume that dramatically negative changes in amylase activity in barley indicate using PAW with RNS, especially NO_2_^−^ and NO_3_^−^ concentrations higher than the beneficial levels for this type of plant and application. A possible mechanism of action may be the signalling activity of NO derived from NO_2_^−^ and NO_3_^−^. In this signal cascade, abundancy of NO inhibits the activity of specific protein kinase (SnRK1), which normally stimulates the activity of amylase in wheat [54,55]. Another mechanism of action may be the inhibition effect of ethanol on gibberellin-induced activity of amylase, as proved by Perata et al. [56], which correlates with our findings on ADH activity (as shown in Section 2.7).

### 2.6. Activity of Antioxidant Enzymes and In Situ Visualisation of Reactive Oxygen Species

**Figure 6:**
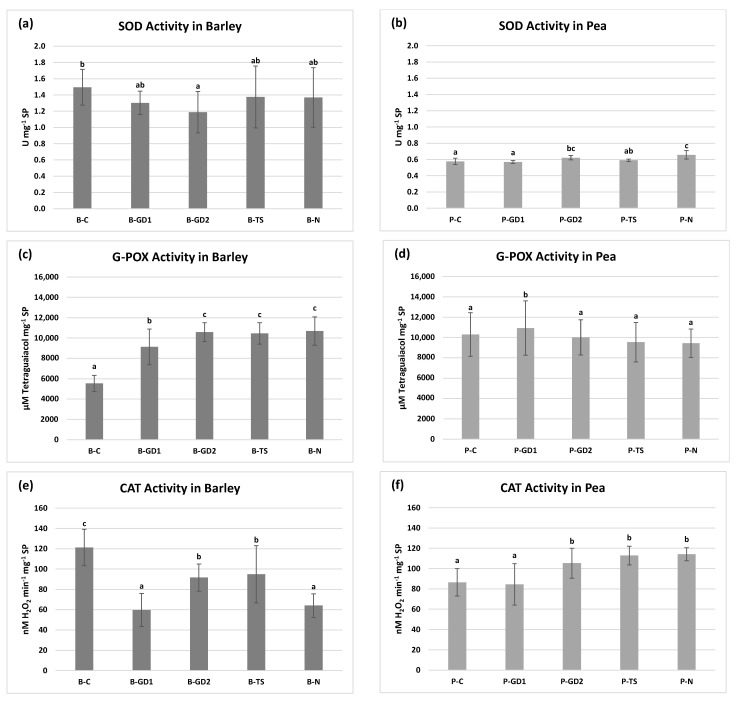
Superoxide dismutase (SOD), guaiacol peroxidase (g-pox) and catalase (CAT) activities in the barley (**a**,**c**,**e**) and pea (**b**,**d**,**f**) seedlings after 3 days of growth, watered with tap water (control: B/P-C), plasma-activated water (PAW) of glow discharge with water cathode and treatment time 1 and 2 min (B/P-GD1 and B/P-GD2), PAW of transient spark discharge with electrospray (B/P-TS) and 2-mM nitric acid (B/P-N). Values are shown as mean ± SD from three experimental rounds, in each round (one run represents five seedlings per variant, 1.5 g mixed samples were analysed per one experimental run and each variant for SOD, G-POX and CAT activity). Different letters represent statistically significant difference at *p* < 0.05 according to LSD test.

**Figure 7:**
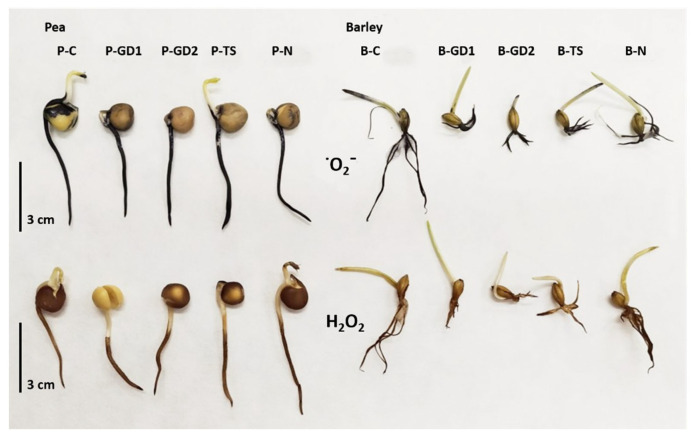
The visualisation of H_2_O_2_ and ˙O_2_^−^ in tissues of the barley and pea seedlings after 3 days of growth, watered with tap water (control: B/P-C), plasma-activated water (PAW) of glow discharge with water cathode and treatment time 1 and 2 min (B/P-GD1 and B/P-GD2), PAW of transient spark discharge with electrospray (B/P-TS) and 2-mM nitric acid (B/P-N). The experiment was repeated four times (one run = five seedlings from each variant were incubated in NBT solution and five seedlings from each variant were incubated in DAB solution, n = 20). The image represents a representative selection of seedlings after staining.

Although PAW is a reactive environment (containing hydrogen peroxide, nitrates and nitrites), it did not cause significant oxidative stress in the case of pea seedlings. In the case of barley, as suggested by other analyses, the situation was moderately different. The activity of SOD, the enzyme that detoxifies the superoxide radicals, slightly decreased in the case of PAW variants of barley compared to the untreated control. In the case of pea seedlings, it increased slightly in the P-GD2 and P-N variants. When monitoring the accumulation of ˙O_2_^−^, we did not notice any significant differences between the individual variants (Figure 6a,b and Figure 7). Our results clearly show that due to the application of PAW, barley and pea seedlings exogenously absorb increased concentrations of H_2_O_2_ (Figure 1b). We can state that barley seedlings reacted negatively to this exogenous application of H_2_O_2_, where we observed a significant shortening of the root length (Figure 3b).

The G-POX activity was significantly increased and CAT activity correlated, and we observed the accumulation of H_2_O_2_ in the whole barley root system and the accumulation of ˙O_2_^−^ in roots as well as in seed coats (Figure 6c,e and Figure 7). The growth and development of pea seedlings were not affected by exogenously applied H_2_O_2_ (Figure 3c). The activity of the enzymes that decompose H_2_O_2_ into water and oxygen was generally comparable to the control. The predominant accumulation of H_2_O_2_ in the root tips and the elongation zone of the roots of pea seedlings is probably associated with intensive cell division and elongation, thus having a physiological basis (Figure 6d,f and Figure 7).

The activity of the antioxidant enzymes, as well as in situ visualisation of oxygen radicals, was a subject of interest also in other studies. In our previous study, we analysed the activity of SOD, G-POX and CAT in 4-week-old barley and maize plants watered with PAW [28]. The results from the barley’s above-ground parts correlate with our current barley data in the activity of SOD and partially with G-POX and CAT activities. However, these findings are in agreement with studies that consider CAT as a dominant enzyme for scavenging H_2_O_2_ in the above-ground parts, while G-POX is considered dominant in the root system of plants [26,57]. Thus, we attribute the significantly higher activity of G-POX and different CAT tendency (in comparison with our previous study [28]) mostly to disparate plant parts that were analysed. In 6-day-old wheat seedlings treated with PAW, Kučerová et al. [26] documented the decrease in activity of all three enzymes in the above-ground parts as well as in the roots. In their other study, Kučerová et al. [21] investigated PAW application on SOD activity in lettuce with the same result. Decreased CAT activity in PAW-treated *Paulownia tomentosa* seedlings was also shown by Puač et al. [58].

On the contrary to the mentioned studies, publications dealing with direct plasma treatment showed mainly a stimulative effect of NTP on antioxidant enzyme activity that could possibly be referred to the different physical factors at play (electric field, UV radiation, short-lived species). For example, Švubová et al. [20,49] showed generally the increased SOD activity in 3-day-old pea seedlings treated with plasma generated in ambient air, oxygen and nitrogen for exposure times varying from 10 s to 300 s. On the other hand, the G-POX activity significantly increased only in variants with nitrogen atmosphere and exposures for 20 s and 40 s, respectively. They associated the ˙O_2_^−^ occurring in root tips of plants treated for 10 s and 20 s with an extensive root growth and development. Higher exposure times than 20 s caused the accumulation of ˙O_2_^−^ additionally in seed coats and cotyledons that the authors attributed to the increased oxidative stress. A similar conclusion has been also obtained in the study of Švubová et al. [59] on 3-day-old soybean seedlings, where exposure times exceeding 60 s induced a severe oxidative stress proven by inefficient decomposing of oxygen radicals.

### 2.7. Activity of Dehydrogenases

**Figure 8:**
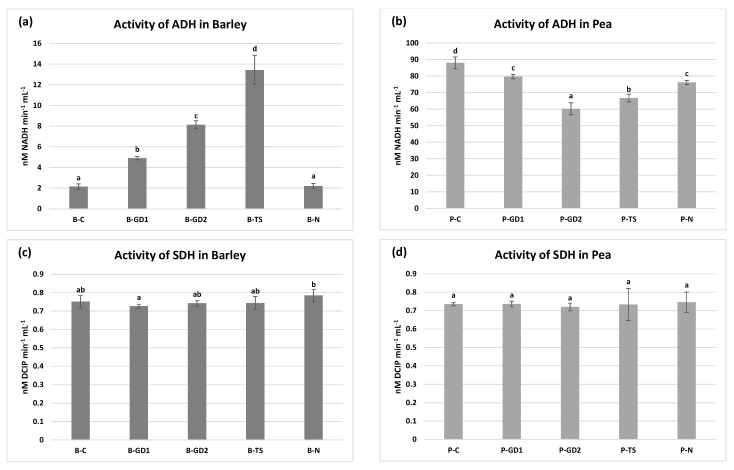
The activity of alcohol (ADH) and succinate (SDH) dehydrogenases in the barley (**a**,**c**) and pea (**b**,**d**) seedlings after 3 days of growth, watered with tap water (control: B/P-C), plasma-activated water (PAW) of glow discharge with water cathode and treatment time 1 and 2 min (B/P-GD1 and B/P-GD2), PAW of transient spark discharge with electrospray (B/P-TS) and 2-mM nitric acid (B/P-N). Values are shown as mean ± SD from three experimental rounds (one run represents five seedlings per variant; five 0.1 g mixed samples were analysed per one experimental run and each variant for SDH activity; five 0.05 g mixed samples were analysed per one experimental run and each variant for ADH activity). Different letters represent statistically significant difference at *p* < 0.05 according to LSD test.

The anaerobic environment during the grain/seed imbibition requires an alternative way to metabolise pyruvate. In this weakly efficient pathway comprising conversion of pyruvate to ethanol, alcohol dehydrogenase (ADH) plays a crucial role. When germination starts, aerobic environment progressively takes control over anaerobic; thus, succinate dehydrogenase (SDH), an important enzyme of the Krebs cycle, becomes dominant in the metabolism of pyruvate [60,61]. Inhibition of SDH leads to retarded germination, blocked hypocotyl elongation and light-dependent seedling establishment as proven by Restovic et al. [62] after using SDH inhibitor (thenoyltrifluoroacetone). Our results indicate that using PAW in barley (Figure 8a) postponed the transition from anaerobic to aerobic conditions, as indicated by high ADH activity (increased ethanol concentration) in all PAW variants. This correlates with decreased amylase activity (Figure 5c) and thus inefficient energy metabolism. The SDH activity in barley, as well as in pea, stayed unchanged (Figure 8c,d). On the other hand, we observed a decrease in ADH activity in pea in all PAW variants and P-N, which refers to an accelerated transition to aerobic metabolism (Figure 8b). This correlates, similar to barley, with the amylase activity that reached its peak in the pea P-GD2 variant (Figure 5d). The studies of Švubová et al. [20,59] showed similar results corresponding with our hypothesis of a postponed turn to aerobic metabolism in the case of barley. In these publications dealing with pea and soybean seedlings, the authors showed a negative impact of high exposure times to direct NTP treatment (with respect to other analyses), resulting in an increase of ADH activity and thus in the suffocation of seeds. Moreover, they documented a positive effect for shorter exposure times that showed an increase in SDH activity simultaneously with a decrease in ADH activity.

### 2.8. DNA Damage

**Figure 9:**
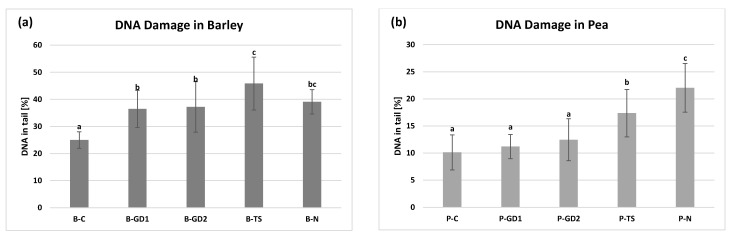
The DNA damage in the barley (**a**) and pea (**b**) seedlings after 3 days of growth, watered with tap water (control: B/P-C), plasma-activated water (PAW) of glow discharge with water cathode and treatment time 1 and 2 min (B/P-GD1 and B/P-GD2), PAW of transient spark discharge with electrospray (B/P-TS) and 2-mM nitric acid (B/P-N). Values are shown as mean ± SD from three experimental rounds. Different letters represent statistically significant difference at *p* < 0.05 according to LSD test.

DNA damage in barley and pea seedlings was analysed using an alkaline comet assay (Figure 9). In pea seedlings, a slight increase in DNA damage was observed in all PAW-treated samples. However, only an increase in the P-TS variant was statistically significant. The highest DNA damage detected in PAW-treated samples was on the level of 17.4% (P-TS variant); however, it could be repaired during plant growth. Damages detected by comet assay are primary and may not have a negative impact on the plant life and plant growth parameters, such as germination (Figure 2) or seedling length (Figure 3). The effect of direct plasma treatment on DNA damage in pea seedlings was analysed in several studies. Švubová et al. [20,49] compared the effect of plasma generated in oxygen, nitrogen or ambient air on pea DNA using the same comet assay. In all plasma-treated samples, an increase in DNA damage was observed in all exposure times (15–300 s). However, none of these damages were caused by double-strand breaks, as was proven by constant field gel electrophoresis. No negative effects of plasma treatment on pea seedlings were observed in Kyzek et al. [63]. In this study, only a slight increase in DNA damage was observed on plasma-treated samples for 1–5 min. Tomeková et al. [64] tried to find the effect of different mixtures of oxygen and nitrogen on DNA damage in pea seedlings. Their results suggest that nitrogen addition to the working gas increased DNA damage in pea seedlings treated with plasma. However, the lowest DNA damage comparable to control samples was observed in seedlings treated with plasma generated in ambient air. Here in the current study, DNA damage in all PAW-treated samples of barley seedlings and B-N variant was statistically significant compared to control samples (B–C).

Higher concentrations of hydrogen peroxide, nitrates and nitrites (Figure 1) could be responsible for the observed damages due to the induction of oxidative stress. All of these three RONS could generate hydroxyl radicals that could easily interact with DNA and cause its damage [65,66,67]. These damages could be repaired, however, only in the initial days of cultivation and could result in lower germination (Figure 2) or root length (Figure 3). Yemeli et al. [28] also analysed DNA damage in barley seedlings after 1-, 2- or 3-week treatment with water treated by the same sources as were used in this study and observed no harmful effects of PAW on barley DNA. However, in this study, younger plants (3-day-old seedlings) were used for experiments, and this could be the reason why the DNA damage was higher (also in control samples). DNA damage in 7-day-old barley seedlings after direct plasma treatment was analysed in the study of Peťková et al. [51]. A statistically significant increase in DNA damage was observed in all samples treated with plasma generated in nitrogen, oxygen or ambient air for 10–60 s. These damages were caused mainly by single-strand breaks and purine oxidation.

## 3. Materials and Methods

### 3.1. Plant Material

Dried barley (*Hordeum vulgare* L.) cv. Kangoo grains and pea (*Pisum sativum* L.) cv. Eso seeds, used as target material in the experiments, were obtained from the Slovenské farmárske družstvo, Slovakia. The pea seeds and barley grains were stored in a fridge at 8 °C in the dark. The experiments were performed in 2020 and 2021.

### 3.2. Experimental Setup, Plasma-Activated Water Production

**Figure 10:**
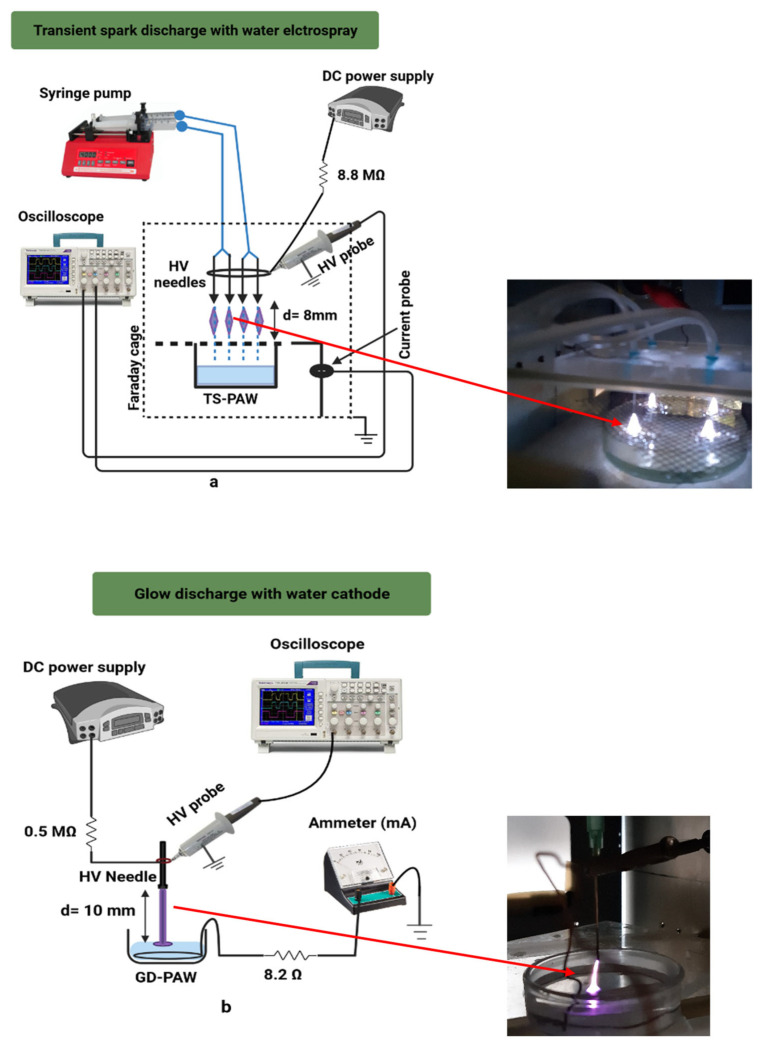
(**a,b**) Schematic diagrams of the experimental setups of TS and GD used in this work to generate PAW with photographs of the TS and GD discharges on the right side.

**Figure 11:**
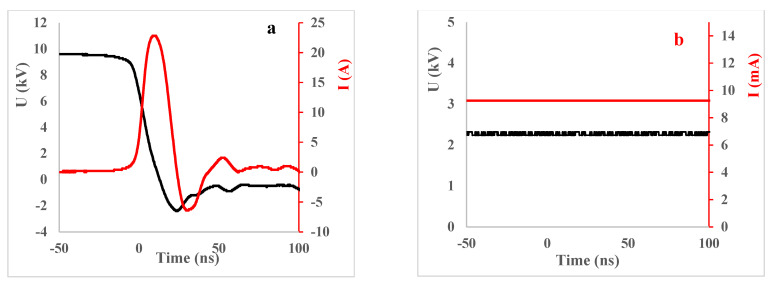
Typical waveforms of the applied discharge voltage and current of (**a**) TS with water electrospray and (**b**) GD with water cathode [28].

Figure 10 and Figure 11 show, respectively, the representation of the experimental setup for plasma water treatments used in this work and the typical waveforms of the applied discharge voltage and current. It also shows the photos of the plasma discharges taken during the activation of water. We used a transient spark (TS) discharge with water electrospray (Figure 10a) described in more detail in our previous articles [28,38,68] and glow discharge (GD) with water cathode (Figure 10b), described in detail in [28,69]. The TS and GD setups contain a high voltage (HV) DC power supply with the following parameters: U_max_ = 20 kV, I_max_ = 30 mA, P_max_ = 600 W. A positive HV is applied directly through the ballast resistors (R = 8.8 MΩ and R = 0.5 MΩ for TS and GD, respectively) on the HV electrode (4 needles for TS and 1 needle for GD, as shown in the setup). The HV probe Tektronix P6015A is used for both setups to measure the voltage. The discharge current is measured for TS plasma by a Rogowski current monitor Pearson electronics 2877 (1 V/1 A) and for GD by the ammeter. The time evolution of electrical parameters of the discharges (voltage and current) is recorded and processed by the digitising oscilloscope Tektronix TDS 2024 (parameters 200 MHz; 2.5 Gs/s; 4 channels).

In this work, tap water was used to produce PAW due to its availability. The discharges are generated in ambient air at atmospheric pressure. For TS, four HV hollow needle electrodes inject the tap water into the active zone of the TS discharge at a constant flow rate 0.5 mL/min per needle by the syringe pump. The treatment time for TS was 25 min to produce 50 mL of PAW used per one seed group in this work. The electrosprayed PAW is collected under the metallic mesh in a Petri dish (see setup). The TS discharge device is held in a Faraday cage due to the electromagnetic radiation.

The generation of PAW by GD was done at a constant activation time of 1 and 2 min. 12 mL of tap water are introduced in a Petri dish containing a metallic wire, and the discharge is initiated between the HV needle electrode and the surface of the water. The water activated by both plasma sources is collected and then subjected to chemical analysis before being used for seed experiments.

### 3.3. Measurement of Nitrites/Nitrates and Hydrogen Peroxide in PAW

The PAW generated were analysed by measuring the pH and the long-life reactive species (hydrogen peroxide, nitrites/nitrates). The RONS detection was performed by UV-Vis absorption spectroscopy colorimetric methods (Shimadzu UV-1800 Spectrophotometer) [70]. The measurement of hydrogen peroxide concentration in PAW was performed by the titanium oxysulfate assay. This colorimetric method is based on the reaction of H_2_O_2_ with the titanyl (IV) ions in strong acidic conditions. The maximum absorption of the yellow-coloured reaction product is at 407 nm. The concentration of NO_2_^−^ and NO_3_^−^ in the PAW was determined by colorimetric assay kit of Griess reagents. This colorimetric method is based on the reaction of nitrites with the Griess reagents, which, after reaction, form a pink-coloured azo product. Nitrates are converted to nitrites by nitrate reductase (with the help of coenzyme) and afterward are analysed the same way as nitrites. Both measurements are done at the maximum absorption at 540 nm. The detailed procedure of the RONS measurement can be found in our previous works [39,40,70]. The following figure shows the representation of the PAW generation and analysis.

### 3.4. Imbibition, Germination and Growth Conditions

Dry pea seeds (50 seeds for each variant) were soaked in 50 mL of tap water (P-C: control) or in PAW (P-TS, P-GD1 and P-GD2 variants) as well as in 2-mM nitric acid (P-N variant) for 1 h at room temperature. The 2 mM HNO_3_ tap water solution was selected to mimic the average nitrate concentration measured in the three types of PAW and represents a positive control providing nitrogen. Imbibed seeds were wrapped in wet sterile filter paper.

Dry barley grains (50 grains for each variant) were sown on Petri dishes (ø 21.5 cm) and watered with 50 mL of tap water (B–C: control) or with PAW (P-TS, P-GD1 and P-GD2 variants) as well as 2-mM nitric acid (B-N variant). Rolls and Petri dishes were cultivated in dark conditions in an incubator at the temperature 24 ± 2 °C for 3 days and fresh tap water or PAW were added each day.

During cultivation, the number of germinated pea seeds/barley grains were counted, and after three days, the material for biochemical analyses was collected (total soluble proteins content, assay on lytic enzymes assessment, assays on antioxidant enzymes and visualisation of ROS, assays on dehydrogenase activities evaluation, comet assay). The length of seedlings (for pea) and length of shoots and roots (for barley) were measured. Percentage of germination was calculated according to Abdul-Baki [71].

### 3.5. Total Soluble Proteins Content Measurement

Samples (~1.5 g) were ground in liquid nitrogen with mortar and pestle and suspended in 50 mM Na-Phosphate protein extraction buffer with 1 mM EDTA, pH 7.8. After 15 min centrifugation (12,000× *g*), the supernatant was used for determination of protein concentration according to Bradford [72]. Total soluble proteins content was calculated as the amount of total proteins per gram of fresh matter from the calibration curve. Bovine Serum Albumin (BSA) was used as a protein standard.

### 3.6. Assay on Lytic Enzymes Assessment

Changes in activity of protease in 3-day-old barley and pea seedlings were determined by incubating 150 µL of an extracted protein sample with 150 µL of 2% (*w*/*v*) BSA in 200 mM glycine-HCl (pH 3.0) at 37 °C for 1 h. The reaction was stopped by the addition of 450 µL of 5% (*w*/*v*) trichloroacetic acid. Samples were incubated on ice for 10 min and centrifuged at 20,000× *g* for 10 min at 4 °C. Absorbance of the supernatant at 280 nm was measured by a spectrophotometer by Jenway 6705 UV/Vis (Bibby Scientific Ltd., Essex, UK) [73].

For determination of α-amylase activities, a commercially-available colorimetric assay kit purchased from Sigma-Aldrich Co. was used. One unit of α-amylase activity is the amount of amylase that cleaves ethylidene-pNP-G7 to generate 1.0 mM of p-nitrophenol per minute at 25 °C.

### 3.7. Assays on Antioxidant Enzymes, Superoxide Dismutase (SOD), Guaiacol Peroxidase (POX) and Catalase (CAT) Activities Assessment and Visualization of ROS (H_2_O_2_ and ˙O_2_^−^)

The activities of enzymes that detoxify ˙O_2_^−^ (SOD, E.C.1.15.1.1) and H_2_O_2_ (POX, E.C.1.11.1.7) were tested. The activity of superoxide dismutase was established according to Beauchamp and Fridovich [74], the guaiacol peroxidase according to Frič and Fuchs [75] and catalase according to Hodges et al. [76]. One unit of SOD activity is the amount of proteins required to inhibit 50% of initial reduction of Nitrotetrazolium Blue Chloride (NBT) under the light. Guaiacol peroxidase activity is expressed in μM of tetraguaiacol min^−1^·mg^−1^ by molar extinction coefficient of tetraguaiacol 26.6. The specific activity of CAT (E.C. 1.11.1.6) was calculated according to Claiborne [77]. Chemicals were purchased from Sigma-Aldrich Co. The presence of H_2_O_2_ and ˙O_2_^−^ was detected in 3-day-old barley and pea seedlings according to Kumar et al. [78]. Hydrogen peroxide was visualised as reddish-brown stain formed by the reaction of 3,3′-Diaminobenzidine (DAB) with the endogenous H_2_O_2_. The content of ˙O_2_^−^ was detected as a dark blue stain of formazan compound, formed as a result of NBT reacting with the endogenous ˙O_2_^−^.

### 3.8. Assays on Dehydrogenase Activities Evaluation

For determination of alcohol (ADH) and succinate (SDH) dehydrogenases in 3-day-old seedlings, commercially-available colorimetric assay kits purchased from Sigma-Aldrich Co. were used. Activities of enzymes were determined according to manufacturer’s instructions. One unit of ADH represents the amount of enzyme that will generate 1.0 mM of NADH per minute at pH 8.0 at 37 °C. One unit of SDH is the amount of enzyme that generates 1.0 µM of 2,6-dichlorophenolindophenol (DCIP) per minute at pH 7.2 at 25 °C.

### 3.9. Comet Assay

Alkaline comet assay is a method used for measuring of DNA damage (single-strand breaks, double-strand breaks, cross-links, apyrimidine and apurine sites) in eukaryotic cells [79,80]. Our experiments were performed according to [20,81]. Briefly, two roots (in the case of pea seedlings) or leaves (in the case of barley seedlings) for each sample were cut by a razor blade in the dark and on ice ensuring DNA release in the 175 µL of 0.4 M Tris-HCl buffer solution (pH 7.5) (Sigma-Aldrich Co., St. Louis, MO, USA). After that, 100 µL of the DNA and buffer suspension was mixed with 100 µL of 1% low melting point agarose (Roth, Karlsruhe, Germany). The final solution was placed on a slide covered by 1% normal melting point agarose (Roth) and then covered by a coverslip. The coverslips were removed after 10 min and the slides were placed in the electrophoretic chamber filled with cold electrophoretic buffer solution containing 1 mM EDTA (Sigma-Aldrich Co.) and 300 mM NaOH (Centralchem, Bratislava, Slovakia) for 8 min. After that, electrophoresis was launched on 1.25 Vcm^−1^ for 15 min at 4 °C. Slides were then neutralised three times with 0.4 M Tris-HCl buffer solution (pH 7.5) and stained with fluorescent dye ethidium bromide (0.05 mM, 80 µL for each slide, Serva, Heidelberg, Germany) for 5 min. DNA damage was observed using fluorescent microscope OLYMPUS BX 51 with green excitation filter UMWIG3 under 400× magnification and evaluated by Comet visual software. Plasma-untreated seedlings were used as negative controls (P-C, B-C).

### 3.10. The Statistical Analysis

The data were analysed using Microsoft Excel (Microsoft Office 365) and Statgraphics Centurion 19 (Statgraphics Technologies, Inc., The Plains, VA, USA). Treatment effects were investigated by means of ANOVA single-step multiple comparisons of means by means of LSD tests, and comparisons between the mean values were considered significant at *p* < 0.05. All experimental data in this work are from at least three independent experiments.

## 4. Conclusions

As shown by many other studies, non-thermal plasma can in certain doses and application methods stimulate plant growth and physiological parameters, besides other positive effects that have been already demonstrated in various life science fields. This study investigated the indirect plasma effect of three types of plasma-activated water (PAW) generated by two different plasma discharges (transient spark with water electrospray and glow discharge with water cathode operated for 1 and 2 min) on 3-day-old seedlings of two important farm plants, barley (*Hordeum vulgare* L. cv. Kangoo) and pea (*Pisum sativum* L. cv. Eso), as a potential alternative to commercial fertilizers supplying plants with nitrogen. Table 1 schematically summarises the obtained results.

PAW applied on pea seeds had a stimulative effect in multiple plant growth and physiological parameters with respect to the negative control (tap water) and positive control (chemically added nitrate in N-variants). Using different types of PAW on pea seedlings exhibits a positive effect on amylase activity and has no inhibition effect on seed germination, seedling length, total protein concentration or protease activity. Moreover, PAW caused no or only moderate oxidative stress, which was in most cases effectively alleviated by natural plant antioxidant enzymes (SOD, G-POX, CAT) and resulted in a very low DNA damage in PAW-treated samples. This effect was also proven by in situ visualisation of H_2_O_2_ and ˙O_2_^−^. In pea seedlings, we observed a faster turn-over from anaerobic metabolism (related to imbibition) to aerobic metabolism, proven by inhibition of alcohol dehydrogenase (ADH) activity. Screening among all variants, the most perspective PAW seems to be the one prepared by the glow discharge at 1 min exposure (variant P-GD1). This PAW contains the lowest concentration of NO_2_^−^ and NO_3_^−^ and an intermediate concentration of H_2_O_2_. With respect to the findings raised from our study and other available ones, we assume that this ratio of RONS could be responsible for effective intracellular signalling, speeding up the transition from anaerobic to aerobic metabolism (proven by inhibition of ADH activity).

Interpreting the results for barley imbibed in three types of PAW was more complex. PAW had no effect on grain germination, total soluble protein and SDH activity when compared to negative as well as positive control. However, after using PAW, we observed the high level of DNA damage together with reduced root and shoot length and decreased amylase activity. These negative effects were attributed to the oxidative stress caused by PAW, which was also exhibited by the enhanced activity of G-POX or ADH related to grain suffocation. Based on other related studies, we can conclude that barley either reacts differently with a delayed positive effect of PAW treatment (that we were not able to record in the early stages of its growth), or this application method combined with the timing does not represent the best way to improve its growth and physiological parameters.

In summary, the use of plasma pre-sowing technologies such as seed/grain imbibition in plasma-activated water seems to be important in the faster recovery of the metabolic activity in grains/seeds (activation of lytic and antioxidant enzymes), if the suitable RONS composition in the PAW is used. In the light of other available studies, it could lead to the faster growth and development of young seedlings and the increase of yield without using chemical fertilizers. Therefore, plasma-activated water exhibits indubitable potential in sustainable and environmentally-friendly agriculture. From the results of this study, we can conclude that concentrations of RONS in GD1 PAW, suitable for faster transition to aerobic metabolism in pea, may not be suitable for another plant species, such as barley, and further investigation needs to be done to answer arising questions on the mechanisms of plant responses to the NTP or PAW treatments.

## Figures and Tables

**Table 1:** An overview of PAW effects on barley (B-) and pea (P-) growth parameters, activity of enzymes and DNA damage. Horizontal arrow represents values without significant change when compared to control plants, upward arrow represents values with significant increase when compared to control plants and downward arrow represent values with significant decrease when compared to control plants.

	B-C	B-GD1	B-GD2	B-TS	B-N	P-C	P-GD1	P-GD2	P-TS	P-N
**Germination**	→	→	→	→	→	→	→	→	→	→
**Root Length**	→	↓	↓	↓	↓					
**Shoot Length**	→	↓	↓	→	→					
**Seedling Length**						→	→	↓	→	→
**TSP**	→	→	→	→	→	→	→	→	→	→
**Protease**	→	→	→	↓	→	→	→	→	→	→
**Amylase**	→	↓	↓	↓	↓	→	→	↑	↑	↓
**SOD**	→	→	↓	→	→	→	→	↑	→	↑
**G-POX**	→	↑	↑	↑	↑	→	↑	→	→	→
**CAT**	→	↓	↓	↓	↓	→	→	↑	↑	↑
**ADH**	→	↑	↑	↑	→	→	↓	↓	↓	↓
**SDH**	→	→	→	→	→	→	→	→	→	→
**DNA Damage**	→	↑	↑	↑	↑	→	→	→	↑	↑
